# Serotonin regulation of behavior via large-scale neuromodulation of serotonin receptor networks

**DOI:** 10.1038/s41593-022-01213-3

**Published:** 2022-12-15

**Authors:** Piergiorgio Salvan, Madalena Fonseca, Anderson M. Winkler, Antoine Beauchamp, Jason P. Lerch, Heidi Johansen-Berg

**Affiliations:** 1 Wellcome Centre For Integrative Neuroimaging, FMRIB, Nuffield Department of Clinical Neurosciences, University of Oxford, Oxford, UK; 2 National Institute of Mental Health, National Institutes of Health, Bethesda, MD USA; 3 Department of Human Genetics, University of Texas Rio Grande Valley, Brownsville, TX USA; 4 Mouse Imaging Centre, The Hospital for Sick Children, Toronto, Ontario Canada; 5 Department of Medical Biophysics, University of Toronto, Toronto, Ontario Canada

**Keywords:** Network models, Functional magnetic resonance imaging, Magnetic resonance imaging, Cognitive neuroscience

## Abstract

Although we understand how serotonin receptors function at the single-cell level, what role different serotonin receptors play in regulating brain-wide activity and, in turn, human behavior, remains unknown. Here, we developed transcriptomic–neuroimaging mapping to characterize brain-wide functional signatures associated with specific serotonin receptors: serotonin receptor networks (SRNs). Probing SRNs with optogenetics–functional magnetic resonance imaging (MRI) and pharmacology in mice, we show that activation of dorsal raphe serotonin neurons differentially modulates the amplitude and functional connectivity of different SRNs, showing that receptors’ spatial distributions can confer specificity not only at the local, but also at the brain-wide, network level. In humans, using resting-state functional MRI, SRNs replicate established divisions of serotonin effects on impulsivity and negative biases. These results provide compelling evidence that heterogeneous brain-wide distributions of different serotonin receptor types may underpin behaviorally distinct modes of serotonin regulation. This suggests that serotonin neurons may regulate multiple aspects of human behavior via modulation of large-scale receptor networks.

## Abstract (web summary)

Combining brain imaging, genetics and behavioral data, this study shows that distinct brainwide distributions of serotonin receptors explain the known division of serotonin effects on human impulsivity and aversive processing.

## Main

Investigating the relationship between large-scale brain activity and behavior can inform us on how a vast array of human behaviors arises from the coordinated activity between neural populations^1,2^. However, neurochemical modulation can bias neural activity by regulating neuronal excitability and plasticity and thus, in turn, affect behaviors. Understanding how neuromodulation orchestrates brain-wide activity is important as it may provide new insight into the regulation of multiple aspects of human behaviors in health and disease.

Serotonin regulates behavior by modulating neuronal excitability and plasticity, and its dysfunction has been implicated in several psychiatric disorders, such as impulsive aggression, anxiety and depression^3^. Serotonin is produced by a surprisingly small proportion of neurons (less than 0.1% of brain neurons) primarily located in the dorsal raphe nucleus (DRN), but it is released widely throughout the brain^4^. In the synaptic cleft, serotonin can interact with multiple receptor types which can vary in spatial distribution, chemical affinities and cellular effects^5,6^. Despite being implicated in a dizzying array of phenomena, a comprehensive theory of how the serotonin system is functionally organized at the macroscopic brain-wide level to support diverse functions remains elusive^7,8^. While the effects of different receptor types at the local level are known, the importance of their brain-wide distribution patterns remains poorly understood. In particular, whether the different spatial patterns of serotonin receptor types provide a macroscale principle of organization for the diverse regulation of human behavior remains unknown.

Historically, serotonin has been associated with both behavioral inhibition^9^ and aversive processing^10^. However, the mechanisms through which serotonin modulates human behavior remain not well understood. On the one hand, serotonin is implicated in impulsive, disinhibited behavior^11–14^; on the other hand, serotonin modulates biases towards aversive processing^8,15^, reflecting negative biases in cognition and behavior that are exacerbated in depression and anxiety. This division of the paradoxical effects of serotonin has been historically explained via the hypothesis that distinct serotonin systems (distinct projections from the DRN and the median raphe nucleus (MRN)) affect diverse neural systems to modulate cognition, affect and behavior^7^. However, recent experimental evidence suggests that even within the DRN functional sub-systems may exist^16^. DRN serotonin neurons have recently been implicated in patience and delayed reward^17–19^, as well as in reward and punishment^16,20–22^. This literature, together with the molecular heterogeneity of the DRN^4^, has suggested the existence of parallel DRN serotonin projections. Recent work using viral–genetic methods has indeed provided strong evidence of the existence of anatomically segregated DRN serotonin projections, with similar responses to reward and opposite response to aversive stimuli^16^. However, this dissection of the DRN serotonin system relies on the concept of anatomical presynaptic segregation, and does not consider the complex, heterogeneous nature of serotonin synapses, characterized by an intricate, complex tapestry of serotonin receptor types^23^.

A prominent view theorizes that neuromodulation regulates different behavioral circuits via different receptor types, with distinct spatial distributions, and with different affinities which endow sensitivity to different timescales and input characteristics^24^. Whilst we understand how serotonin receptors function at the single-cell level^23^, we do not yet understand how these receptors affect brain-wide activity and, in turn, human behavior. This is fundamental to better understand serotonin’s role both in health and in disease.

To understand how different serotonin receptors shape serotonin regulation of brain-wide activity and affect behavior requires consideration of both spatio-temporal dynamics of serotonin neuromodulation and variation in human behavior. Neuroimaging provides measurements that are sensitive to cellular phenomena and that can also be acquired in living humans, allowing us to bridge between cellular mechanisms investigated in animal models and human population variation in brain and behavior. Here, we combined neuroimaging and gene expression maps to extract brain-wide functional signatures associated with specific serotonin receptors to investigate how different serotonin receptor types regulate brain-wide activity and, ultimately, behavior. First, by combining optogenetics, pharmacology, whole-brain imaging and gene expression maps in mice, we test whether distinct brain-wide networks, characterized by different serotonin receptor types (SRNs), are differentially modulated by serotonin manipulations. Next, using the same neuroimaging phenotypes in humans, we ask whether variability in SRNs can account for population variation in human mental processes previously implicated in serotonin function. This work provides a mechanistic understanding of how DRN serotonin actions on different serotonin receptor types may mediate the regulation of distinct aspects of human behaviors.

## Results

### Mapping spatio-temporal functional MRI signatures of brain-wide serotonin receptors

**Fig. 1:**
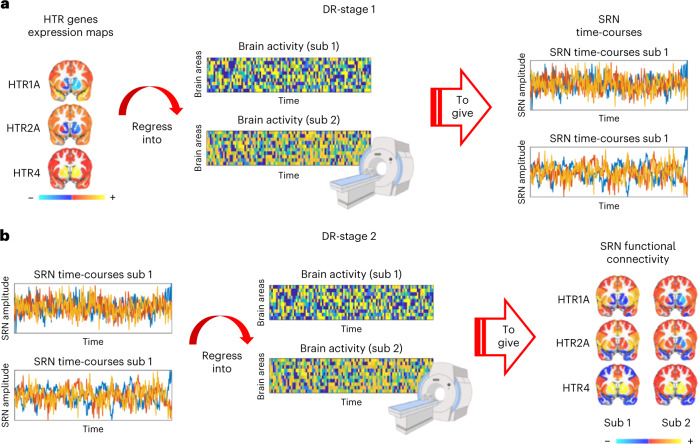
Mapping functional signatures of brain-wide SRNs. Gene expression brain maps of serotonin receptor genes are combined with fMRI (both mouse and human) via FSL DR to map temporal and spatial fMRI signatures of different serotonin receptor types. **a**, DR-stage 1: regresses the spatial maps of serotonin receptor genes into each subject’s four-dimensional (4D) fMRI dataset. This gives a subject-specific time-course quantifying network amplitude changes for each SRN. DR-stage 1 can be used to address questions such as: What are the SRN amplitude changes in response to optogenetic stimulation? **b**, DR-stage 2: regresses subject-specific time-courses into the same 4D fMRI dataset. This provides a subject-specific spatial map quantifying functional connectivity for each SRN. DR-stage 2 can be used to address questions such as: What are the SRN brain correlates associated with individual differences in a depression scale? Sub, subject.

To determine functional signatures associated with different serotonin receptors we developed a transcriptomic–neuroimaging approach that combined mouse and human gene expression brain maps from the Allen Institute with functional MRI (fMRI) (Fig. 1). Using optogenetics–fMRI (ofMRI) data in mice and resting-state fMRI (rs-fMRI) data in humans, we characterized spatio-temporal fMRI signatures associated with specific serotonin receptors. We refer to these signatures as SRNs.

**Extended Data Fig. 1:**
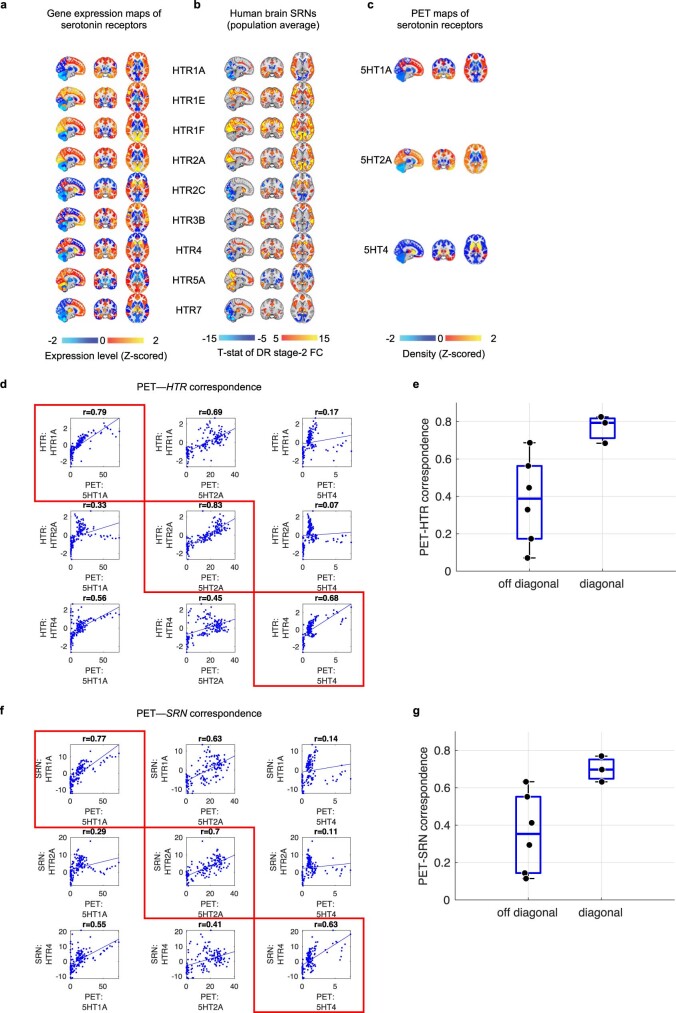
Spatial correspondence between gene expression maps, SRNs, and PET maps. Related to Fig. 1. **a**) Showing human brain maps (rank-based inverse normalised) of serotonin receptor genes publicly available from the Allen Brain Institute. **b**) Showing group-level maps (T-stats) of human brain SRNs functional connectivity (DR-stage 2) estimated by combining transcriptomic maps (displayed in panel **a**) with resting state fMRI data from the Human Connectome Project using FSL DR as exemplified in Fig. 1. **c**) Publicly available serotonin receptor density maps previously characterised using PET. (Note, only maps for receptors 5-HT1A, 5-HT2A, and 5-HT4, were available. Here maps are Z-scored for display purposes). **d**) Spatial PET—HTR correspondence between maps of serotonin receptor density previously characterised using PET (displayed in panel **c**) and human gene expression maps of serotonin receptors (displayed in panel **a**). Spatial similarity was calculated using Pearson’s correlation across atlas brain regions. Red squares highlight diagonal elements of the correlation matrix. **e**) Boxplot showing difference in PET—SRN correspondence between diagonal and off diagonal elements of panel **d**). **f**) Spatial PET—SRN correspondence between maps of serotonin receptor density previously characterised using PET (displayed in panel **c**) and human SRN functional connectivity maps (displayed in panel **b**). Spatial similarity was calculated using Pearson’s correlation across atlas brain regions. Red squares highlight diagonal elements of the correlation matrix. **g**) Boxplot showing difference in PET—SRN correspondence between diagonal and off diagonal elements of panel **d**). In **e**) and **g**), on each box, the central mark indicates the median, and the bottom and top edges of the box indicate the 25th and 75th percentiles, respectively. The whiskers extend to the most extreme data points not considered outliers. Each data point is plotted individually (n = 9 maps). Together panels **e**) and **g**) show greater PET—HTR and PET—SRN spatial correspondence for the same serotonin receptors (diagonal elements) compared with the spatial correspondence between different serotonin receptors (off diagonal elements). FC: functional connectivity.

We leveraged publicly available mouse and human brain transcriptomic maps of serotonin receptor genes (*HTR1-7* (refs. ^25,26^)). Each map characterizes the brain-wide gene expression level for a single serotonin receptor gene. We used the established tool FMRIB Software Library (FSL) Dual Regression (DR)^27^ to relate these gene expression maps to fMRI. For each subject, FSL DR first computes an SRN-specific time-course (reflecting the amplitude of network activity; DR-stage 1; Fig. 1a) and then a functional connectivity spatial map (reflecting the spatially distributed nature of correlations; DR-stage 2; Fig. 1b). FSL DR-stages 1 and 2 thereby provide temporal and spatial fMRI signatures of different serotonin receptor genes, respectively. Greater correlation in fMRI time-courses in brain areas with similar gene expression levels will result in greater SRN amplitude changes and functional connectivity. Furthermore, because FSL DR is a multivariate regression approach^27^, these signatures are unique to each receptor. At the group level, human *HTR* genes and SRN functional connectivity maps showed a good spatial correspondence with group-level serotonin receptor density maps independently characterized via positron-emission tomography (PET)^28^ (Extended Data Fig. 1). Importantly, this transcriptomic–neuroimaging mapping approach can be equally applied to mice ofMRI data and human rs-fMRI data, thus providing translatable neuroimaging phenotypes.

### Optogenetics of mouse DRN serotonin neurons elicits different changes in SRNs

**Fig. 2:**
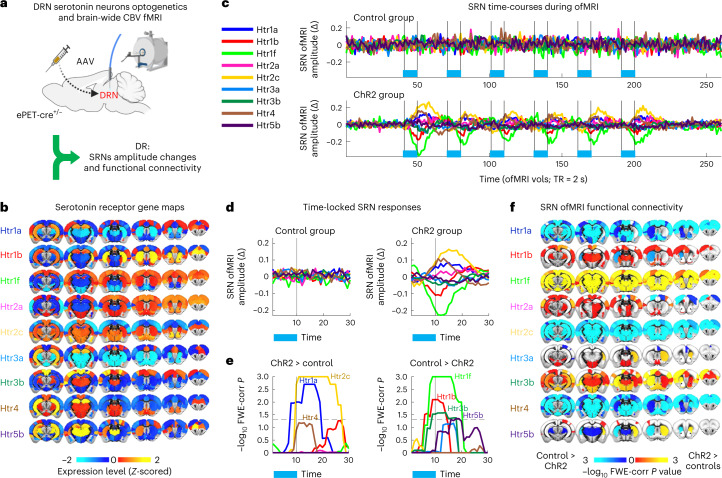
Activity of DRN serotonin neurons modulates SRNs. **a**, Mice were genetically manipulated to express ChR2 in DRN serotonin neurons before ofMRI experiments^29^. **b**, Transcriptomic maps of serotonin receptor genes from the Allen Brain Institute. The combination of serotonin receptor maps and ofMRI via FSL DR allowed us to characterize SRNs. **c**, SRN time-courses of amplitude changes (DR-stage 1) during ofMRI. (Δ, changes from baseline activity; see Extended Data Fig. 2a). Top row shows SRN amplitude changes for control animals; bottom row for ChR2 animals. Each color represents an SRN; SRN colors are matched with the other panels. **d**, Time-locked ofMRI amplitude changes of SRNs (DR-stage 1) in control animals (left) and in ChR2 animals (right). Blue bar underneath represents when optogenetic stimulation was on. **e**, Results from permutation analysis of linear models testing differences between control and ChR2 animals on SRN time-locked responses. Statistical significance was assessed with 1,000 block-aware permutations, with FWE-corr for multiple comparisons across time, SRNs and two tails. Showing −log_10_ FWE-corr *P*, corrected across time, SRNs and two tails. Dashed lines demarcate statistical thresholds. Together, panels **d** and **e** show a heterogeneity of SRN amplitude changes in response to optogenetic activation of DRN serotonin neurons. **f**, Results from permutation analysis of linear models testing differences between control and ChR2 groups on SRN functional connectivity maps (DR-stage 2). Statistical significance was assessed with 1,000 block-aware permutations, with FWE-corr for multiple comparisons across brain regions, SRNs and two tails. Showing −log_10_ FWE-corr *P*, corrected across brain regions, SRNs and two tails. Cold colors represent decreased functional connectivity in the ChR2 group compared with the control group; hot colors represent increased functional connectivity. The results of this figure demonstrate that the spatial distribution of different serotonin receptor types confers functional specificity at the brain-wide level. CBV, erebral blood volume; TR, repetition time; vols, volumes.

**Extended Data Fig. 2:**
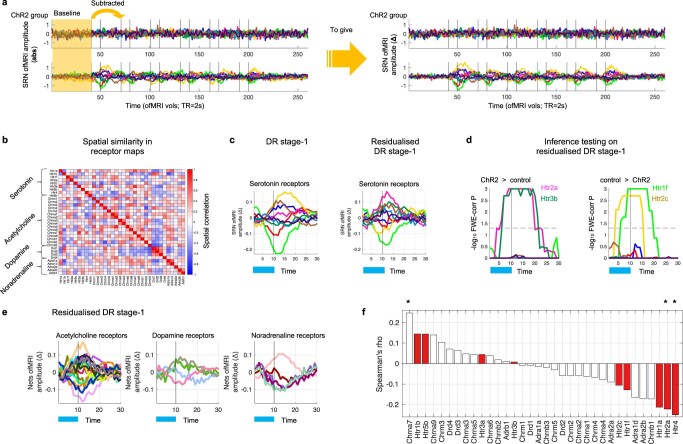
SRNs fluctuations during DRN ofMRI and specificity. Related to Fig. 2. **a**) Left: Showing SRN fluctuations in absolute (abs) amplitude in response to DRN ofMRI. Yellow area indicates the average SRN absolute (abs) amplitude activity (DR-stage 1 SRN amplitude fluctuations) during the first 40 timepoints. This average activity was subtracted from the remaining timepoints in order to take into account baseline. Right: Showing SRN delta (Δ) amplitude obtained from the process depicted in the left. **b**) Spatial similarity between all receptor maps: serotonin, acetylcholine, dopamine, and noradrenaline, demonstrating that there is low to modest correlation between most spatial maps as expected, as different receptor maps have distinct though overlapping spatial distributions. Spatial similarity was calculated at the voxel-wise whole-brain level. **c**) Time-locked ofMRI amplitude changes in SRNs calculated in ChR2 animals (left) via DR stage-1 (as also shown in main figures) and (right) via *residualised* DR stage-1. Blue bar underneath represents when optogenetic stimulation was on. **d**) Results from permutation analysis of linear models on *residualised* DR stage-1 SRN time-locked responses between control and ChR2 animals. Statistical significance was assessed with 1,000 block-aware permutations with FWE-corr for multiple comparisons. Showing -log10 FWE-corr p-values, corrected across time, SRNs, and two tails. Dashed lines demarcate statistical thresholds. **e**) Time-locked ofMRI amplitude changes in other receptor networks (Nets) calculated in ChR2 animals via *residualised* DR stage-1. **f**) Spearman’s partial correlation between multiple neuromodulatory receptor maps and DRN ofMRI activity as previously implemented by Zerbi et al. (see Methods). Two-tailed significance was assessed with permutation testing and partial correlations were deemed significant at FWE-corr P < 0.025. The * symbol above bars indicates a statistically significant FWE-corr p-value. Red bars indicate serotonin receptors. This analysis shows that serotonin receptors have the highest partial correlation values with DRN ofMRI changes.

Given the different spatial distribution and response properties of different receptors, it is plausible that the same serotonin manipulation will differently modulate distinct SRNs. Here, in mice, we tested the hypothesis that optogenetics activation of DRN serotonin neurons elicits distinct changes in brain-wide SRNs. We used existing publicly available data from an ofMRI manipulation of ePet-Cre mice expressing channelrhodopsin-2 (ChR2) in DRN serotonin neurons (Fig. 2a)^29^, and re-analyzed them with the transcriptomic–neuroimaging approach described above. Whilst ofMRI allows for precise causal manipulation of DRN serotonin neurons and concurrent recording of brain-wide function, transcriptomic–neuroimaging mapping of serotonin receptor genes (*Htr1-5*; Fig. 2b; ref. ^25^) allows us to determine how different SRNs contribute to the brain-wide effect of activating DRN serotonin neurons. Using transcriptomic–ofMRI mapping, we can extract one time-series (Fig. 2c–e and Extended Data Fig. 2a) and one brain-wide functional connectivity map (Fig. 2f) for each receptor gene, for each experimental mouse. Importantly, because FSL DR is a multivariate regression approach^27^ and transcriptomic–neuroimaging mapping is performed by regressing all serotonin receptor maps in the same regression model, the estimated temporal and spatial signatures are unique to each serotonin receptor map included in the analysis.

First, we investigated whether 20 s of optogenetic stimulation of DRN serotonin neurons at 20 Hz causes amplitude fluctuations in SRNs. Although the DRN system is known to co-release glutamate, prolonged DRN stimulation at 20 Hz is known to have predominant serotonergic effects rather than glutamate-mediated^22,30^. Here, in ChR2 mice, we observed that activating DRN serotonin neurons gave rise to a variety of time-locked network amplitude changes (output from DR-stage 1) in SRNs (Fig. 2d). These network amplitude changes were not present in control mice. Using permutation-based inference testing (Methods), we found that whilst *Htr2c* and *Htr1a* SRN amplitude changes significantly increased due to optogenetic stimulation compared with controls, *Htr3a* and *Htr3b* responses significantly decreased (Fig. 2e). This dichotomy between the pairs *Htr1a*-*Htr2c* and *Htr3a*-*Htr3b* is particularly interesting as 5-HT3 receptors are the only ionotropic receptors among all 14 serotonin receptors. We also found that *Htr1b* SRN amplitude changed over time (initial significant decrease followed by a significant increase), that *Htr5b* SRN amplitude changes showed a delayed increase and that the *Htr4* showed an increase in amplitude. These results show a heterogeneity of SRN temporal amplitude changes in response to activation of DRN serotonin neurons.

Second, we assessed the specificity of serotonin receptors alone to explain DRN ofMRI fluctuations. Specificity is initially achieved because DRN serotonin ofMRI, by genetically targeting serotonin neurons, experimentally guarantees a priori specificity in downstream regulation. In the main analysis we used transcriptomic–neuroimaging mapping to investigate fluctuations in SRNs mediating serotonin downstream modulation. To test whether the reported effects are specifically related to serotonin receptors alone, we performed transcriptomic–neuroimaging mapping whilst co-varying for non-serotonin receptor maps. We used non-serotonin receptor maps belonging to other neurochemical modulators (acetylcholine, dopamine, noradrenaline) as confound regressors. In this way, we investigated fluctuations in nine serotonin receptor maps (our original SRNs) in response to serotonin neurons ofMRI after adjusting for 25 non-serotonin receptor maps. We refer to this approach as residualized DR-stage 1. By regressing out spatial variance shared with non-serotonin maps, this approach provides only ofMRI signatures that are unique to serotonin receptors. As evident from the results of this analysis (Extended Data Fig. 2b), we found DRN serotonin ofMRI responses that are unique to serotonin receptors alone, even after accounting for 25 non-serotonin receptor maps. Furthermore, such fluctuations are significantly different between ChR2 and controls (Extended Data Fig. 2c,d). Although some SRN responses changed in amplitude or sign compared with the main results—this is most likely due to the collinearity between serotonin and non-serotonin maps—these supplementary results are crucially aligned with the main results: they show that serotonin receptors alone can explain heterogeneity in serotonin downstream modulation. It is important to highlight, however, that the specificity of our method to study SRN fluctuations is limited by the receptor co-expression across neuromodulators. This is a key property of the brain which allows multiple neuromodulators to act on the same functions^24^. Indeed, other receptors also show changes in response to DRN serotonin ofMRI (Extended Data Fig. 2e), suggesting a chain of events which released other neuromodulators. Nevertheless, when specificity is assessed using a previously established alternative method^31^, results show specificity for serotonin receptors in DRN ofMRI (Extended Data Fig. 2f). These results demonstrate that SRNs retained specificity to explain heterogeneity in serotonin downstream modulation despite co-expression of multiple receptors across neuromodulators.

Third, we investigated the SRN spatial correlates (DR-stage 2) of DRN serotonin activation. Using permutation-based inference testing, we found that activation of DRN serotonin neurons could either increase or decrease functional connectivity, either locally or globally (Fig. 2f), independently of whether an SRN temporal response increased or decreased in amplitude. For example, whilst a significant temporal decrease in the *Htr3a* SRN amplitude resulted in decreased functional connectivity across brain regions, a similar decrease in temporal amplitude in the *Htr1b* SRN resulted in increased functional connectivity. These results show that different brain areas, depending on the admixture of serotonin receptors they express, can show radically different fMRI responses to DRN serotonin neuron activation. Whilst heterogeneous responses would be expected, given the different cellular effects of different receptors, our non-invasive approach allows us to demonstrate how such differences play out across time, and throughout the brain, providing a unique window into brain-wide receptor-specific dynamics. In addition, these results raise the possibility that the heterogeneous spatial distributions of serotonin receptor types may reflect not only a way for a single neurotransmitter to differently influence different local circuits, but also a more global mechanism to coordinate activity within defined large-scale networks. In other words, a macroscale principle of organization of serotonin neuromodulation.

### Fluoxetine manipulation of brain-wide SRN responses to DRN ofMRI

**Fig. 3:**
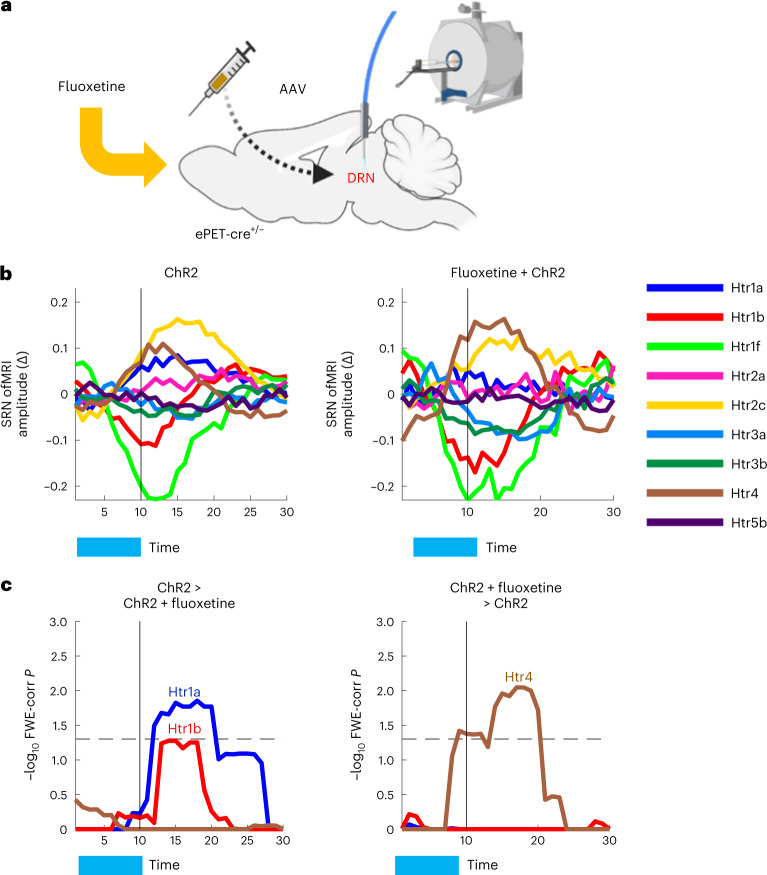
Fluoxetine manipulation alters neuromodulation in predicted SRNs. **a**, ChR2 animals were treated with one pharmacologically significant dose of fluoxetine and then underwent ofMRI. **b**, Time-locked ofMRI amplitude changes of SRNs in ChR2 animals (left) and in ChR2 animals treated with fluoxetine (right). Each color represents an SRN. **c**, Results from permutation analysis of linear models testing for group differences in *Htr1a*, *Htr1b* and *Htr4* SRN time-locked amplitude changes calculated via DR-stage 1, between the ChR2 group and the ChR2 group treated with fluoxetine. Statistical significance was assessed with 1,000 block-aware permutations (whilst allowing permutations only within-subject), with FWE-corr for multiple comparisons across time, SRNs and two tails. Showing −log_10_ FWE-corr *P*, corrected across time and two tails. Dashed lines demarcate statistical thresholds. These results show that known pharmacological effects on serotonin receptor types can be detected with our approach, hence providing an important validation, and that the spatial distribution of different serotonin receptor types confers functional specificity when selectively manipulating serotonin availability in synaptic terminals.

**Extended Data Fig. 3:**
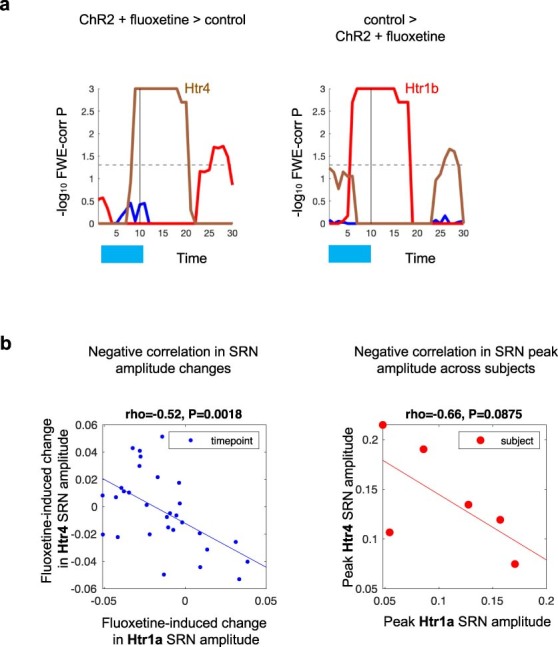
Cross-receptors relationship between *Htr1a* and *Htr4* SRNs. Related to Fig. 3. **a**) Results from permutation analysis of linear models for group differences between the ChR2 group treated with fluoxetine and the control group in SRN time-locked amplitude changes. Statistical significance was assessed with 1,000 block-aware permutations (whilst allowing permutations only within-subject), with FWE-corr for multiple comparisons. Showing -log10 FWE-corr p-values, corrected across time and two tails. Dashed lines demark statistical thresholds. **b**) Showing negative correlations between *Htr1a* and *Htr4* SRN activity. Correlation assessed via one-tail Spearman’s correlation. Left: scatter plot relating fluoxetine-induced changes in *Htr1a* and *Htr4* SRN amplitude during ofMRI experiments. Right: scatter plot relating fluoxetine-induced peak activity in *Htr1a* and *Htr4* SRN amplitude across subjects. For both left and right scatter plots, fluoxetine-induced changes are *fluoxetine* + *ChR2* minus *ChR2 only* ofMRI amplitude changes.

To better understand serotonin regulation of SRNs and further validate our approach, we then asked how selectively manipulating serotonin availability in synaptic terminals modulates SRNs. We took advantage of fluoxetine, a selective serotonin reuptake inhibitor (SSRI), which is known to alter synaptic serotonin availability and is used as a major therapeutic option for psychiatric disorders. Although the effects of fluoxetine on brain activity are not completely understood, previous research suggests that certain serotonin receptor types may be particularly important for its therapeutic effects. The acute administration of SSRIs is known to indirectly activate 5-HT1 receptors and, in turn, to inhibit DRN serotonin cell firing and to decrease extracellular serotonin as measured via in vivo microdialysis^32^. Furthermore, previous work has shown that 5-HT4 receptor activation is necessary for the effects of fluoxetine^23,33^. Here, we leveraged our transcriptomic–neuroimaging mapping approach described above to study the effect of fluoxetine on SRNs. We made use of a publicly available dataset^29^, in which an acute, pharmacologically relevant dose of fluoxetine (4.5 mg kg^−1^ (ref. ^34^)) was administered via tail vein infusion during ofMRI (Fig. 3a). Using this dataset, we tested the hypothesis that fluoxetine significantly changed DRN modulation of the *Htr1a*, *Htr1b* and *Htr4* SRNs. Using permutation-based inference testing, we contrasted on/off fluoxetine within-subject ofMRI runs in ChR2 animals. Surprisingly, we found a bidirectional effect of fluoxetine: whilst fluoxetine downregulated DRN modulation of *Htr1a* and *Htr1b* SRNs’ amplitude response, it upregulated DRN modulation of the *Htr4* SRN (Fig. 3b,c; although these effects did not significantly alter SRNs’ functional connectivity; results not shown). Interestingly, we also found that fluoxetine DRN manipulation of *Htr1a* SRN did not significantly differ from controls (Extended Data Fig. 3a). We then took further advantage of the fluoxetine–ofMRI set-up to investigate the relationship between *Htr1a* and *Htr4* as influenced by fluoxetine–ofMRI. Previous research has indeed hypothesized that the interaction between these two receptor types may be at the basis of a negative feedback control loop^35^ influencing serotonin neuromodulation. We found a negative correlation between *Htr1a* and *Htr4* SRNs, both across amplitude changes and across subjects (Extended Data Fig. 3b). These results show that changes in serotonin availability in synaptic terminals during DRN ofMRI may directly modulate interactions between areas expressing *Htr1a* and *Htr4* receptors.

The results are supported by previous studies showing that 5-HT1A receptor antagonists prevent the acute inhibitory effect of SSRIs on DRN cell firing, thus augmenting the effect of fluoxetine^36,37^. The results are also aligned with previous literature showing that serotonin 5-HT4 receptor-mediated synaptic potentiation plays a central role in fluoxetine’s antidepressant actions^38^. Furthermore, whilst short-term treatment via 5-HT4 receptor agonists mimics the anxiolytic/antidepressant-like effects achieved after chronic fluoxetine administration, 5-HT4 receptor antagonists block these effects^33^, demonstrating a key role for this pathway. These results show that combining transcriptomic maps with pharmacological–ofMRI can capture spatio-temporal effects known to exist in the current literature, and therefore provide a strong validation of the mapping approach described here to study serotonin neuromodulation and the role of different receptor types. Furthermore, these results demonstrate that combining our approach with precise causal manipulations can be leveraged to unravel receptor-specific brain mechanisms of drug effects in a temporally dynamic manner.

### Human mental processes are organized into independent modes of SRN modulation

Serotonin has been implicated in a myriad of cognitive and behavioral functions, yet a comprehensive understanding of how this is achieved at the brain-wide level is still lacking. The existence of multiple serotonin receptors with different cellular effects offers a mechanism by which a single neuromodulator can have diverse downstream effects. Yet, how this palette of receptors is used at the whole-brain level to regulate different behaviors remains a mystery.

Building on the transcriptomic–neuroimaging mapping approach developed above, here we studied the link between human brain functional organization of SRNs and human behavior at the population level. Using data from the Human Connectome Project (HCP)^39^ and by integrating hypothesis- and data-driven approaches, we tested whether differences in SRN functional connectivity could account for individual differences in specific mental processes previously implicated in serotonin function, such as delayed reward discounting^11^, impulsivity and flexibility^7,14^, reward^15^ and punishment^16^, episodic memory^40^, affect, personality and social behavior^13^, as well as depression and anxiety measures^41,42^. Importantly, here, we tested the hypothesis that admixtures of serotonin receptors (as opposed to single receptors) are related to inter-subject differences in human behavior.

**Fig. 4:**
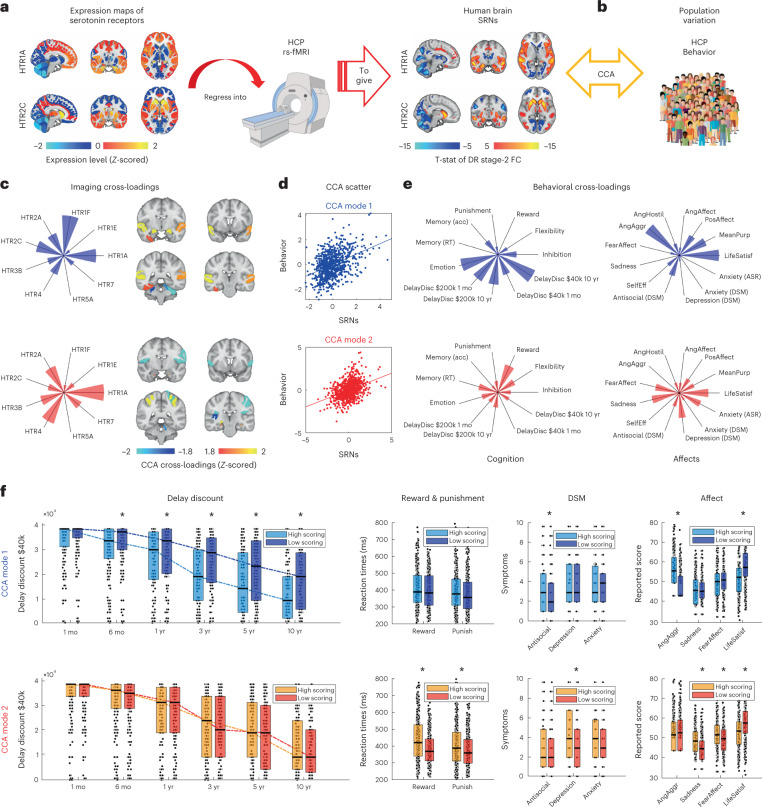
Human mental processes are organized into independent modes of SRN modulation. **a**, FSL DR approach to derive SRNs in the HCP dataset. **b**, Individual differences in SRNs were tested for covariation with behaviors via CCA. Permutation inference CCA characterized two statistically significant orthogonal modes of population variation. Each mode captures a relationship between SRN functional connectivity measures (‘imaging cross-loadings’) and behavioral measures (‘behavioral cross-loadings’). Modes 1 and 2 are color-coded in blue and red, respectively, throughout the main figure and the related figure (Extended Data Fig. 4). **c**, For each mode, imaging cross-loadings are represented as a set of brain regions showing a specific fingerprint of SRN involvement (radial plot, where each axis represents a receptor type). (For un-thresholded imaging cross-loadings, see Extended Data Fig. 4a–c.) **d**, Scatter plots of significant CCA modes: top: CCA mode 1, *r* = 0.42; FWE-corr *P* = 0.0008; bottom: CCA mode 2, *r* = 0.39; FWE-corr *P* = 0.0253. Statistical significance assessed via 10,000 block-aware permutations and FWE-corr for multiple comparisons. The scatter plots illustrate the relationship between SRNs and behavior. **e**, For each mode, behavioral cross-loadings are represented as axial plots illustrating fingerprints of cognitive (left) and affective (right) variables, where each axis represents a behavior score (see Extended Data Fig. 4d for fingerprints on the full set of behavioral variables). **f**, For each mode, subjects were ranked according to their CCA subject score (which captures how well individuals are represented by the mode phenotype) and divided into ‘high scoring’ and ‘low scoring’ groups. Behavioral scores were then calculated for these two groups separately, for measures of delay discounting, reward and punishment, psychiatric scores (according to the Diagnostic and Statistical Manual of Mental Disorders (DSM)) and affect, to test whether the modes of variation identified by the CCA captured relevant differences in behavior in these domains. On each box, the central mark indicates the median, and the bottom and top edges of the box indicate the 25th and 75th percentiles, respectively. Each data point represents an individual (*n* = 812 individuals). *Significant difference between high- versus low-scoring group (one-way analysis of variance; Bonferroni corrected across two CCA modes and multiple variables within the domain of interest). The results in panel **f** show that while mode 1 primarily captures variance in delay discounting (*P* < 10^−4^), antisocial problems (*P* < 10^−4^) and anger/aggression (*P* < 10^−4^), mode 2 captures variation in reward (*P* < 10^−4^) and punishment (*P* = 0.0071), depression (*P* < 10^−4^) and panic/sadness (*P* < 10^−4^). Both modes showed a significant effect on life satisfaction (both, *P* < 10^−4^). FC, functional connectivity; T-stat, T-statistic.

To do this, we combined human brain transcriptomic maps of serotonin receptor genes (*HTR1-7*) with resting-state blood oxygenation level-dependent (BOLD) fMRI data from 812 subjects^1^ using the FSL DR approach described above (DR-stage 2) (Fig. 4a). This allowed us to quantify subject-specific functional connectivity strength for each SRN, thus characterizing individual differences in the brain-wide functional organization of SRNs. Forty-five non-imaging variables (Supplementary Table 1), measuring cognition, behavior, affect and psychiatric symptoms, were then considered for SRNs–behavior covariation analysis (Fig. 4b).

**Extended Data Fig. 4:**
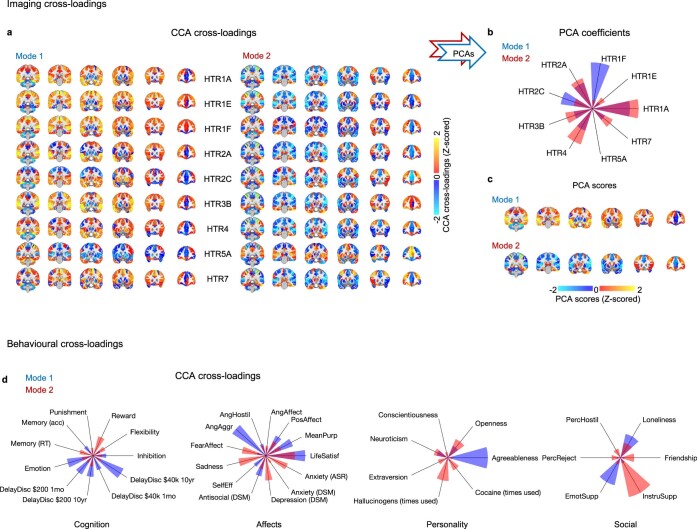
Full set of imaging and behavioural CCA cross-loadings. Related to Fig. 4. **a**) For each CCA mode, showing non-thresholded brain maps of imaging cross-loadings. As the CCA was carried out on ROI-based SRN functional connectivity values, maps are displayed preserving this level of resolution. To ease visualisation and interpretation, separately for each mode, brain maps of imaging cross-loadings (panel **a**; brain regions–by–SRNs) were fed to a PCA (extracting only one single component a max variance). This resulted in **b**) a set of PCA coefficients (SRN fingerprint; also shown in Fig. 4c) and in **c**) PCA scores (brain map; also shown thresholded in Fig. 4e). **d**) For each CCA mode, showing the full set of behavioural cross-loadings.

Using canonical correlation analysis (CCA), we investigated modes of population covariation between SRNs and mental processes previously implicated in serotonin function, and assessed their statistical significance using permutation methods. Briefly, CCA characterizes covariation modes between two sets of variables (here, SRNs and behavior) in the form of pairs of latent projections (CCA covariates) that are maximally correlated. In other words, each covariation mode (Fig. 4d) links a pattern of SRN functional connectivity (Fig. 4c) with a behavioral phenotype (Fig. 4e) across individuals. Here, we found two statistically significant modes of population variation linking distinct sets of SRNs with distinct phenotypes of human mental processes previously implicated in serotonin function (Fig. 4c–e and Extended Data Fig. 4). Importantly, CCA modes are by construction orthogonal to each other, and hence CCA mode 1 captures inter-subject differences in population variation that are independent to those captured by CCA mode 2.

Across individuals, CCA mode 1 related greater *HTR1A*, *HTR1F* and *HTR2C* SRN functional connectivity in the amygdala and temporal cortex, with greater impulsivity in intertemporal choice (during delayed reward discounting task) (Fig. 4f, top row), greater levels of antisocial problems (*Antisocial DSM* HCP-variable) and greater emotional levels of aggression (*AngAggr DSM* HCP-variable). CCA mode 2 instead related greater *HTR1A* and *HTR4* SRN functional connectivity in the parietal cortex and rolandic operculum, with slower responsiveness to reward (during gambling decision making task) (Fig. 4f, bottom row), greater levels of depression (*Depression DSM* HCP-variable; as well as greater levels of self-reported anxiety; *Anxiety ASR* HCP-variable), and greater emotional levels of panic (*FearAffect* HCP-variable) and sadness (*Sadness* HCP-variable).

Together, these results show two modes (or latent factors) of SRN modulation which are independent from each other, load differentially into distinct SRNs, localize in different brain areas and, importantly, have distinct phenotypes of mental processes implicated in serotonin function. The first mode captures a phenotype that is related to impulsivity (or impatience in intertemporal choice), antisocial problems and anger/aggression. The second mode is related to a negative bias in reward processing, depression and panic/sadness. These results replicate and hence explain the established division of the effects of serotonin on human behavior^7^. They provide evidence for how ‘paradoxical’ effects of serotonin on human behavior, behavioral inhibition and aversive processing, respectively^7^, can be explained via complementary modes of SRNs modulation arising from the complex tapestry of postsynaptic serotonin receptor types.

**Extended Data Fig. 5:**
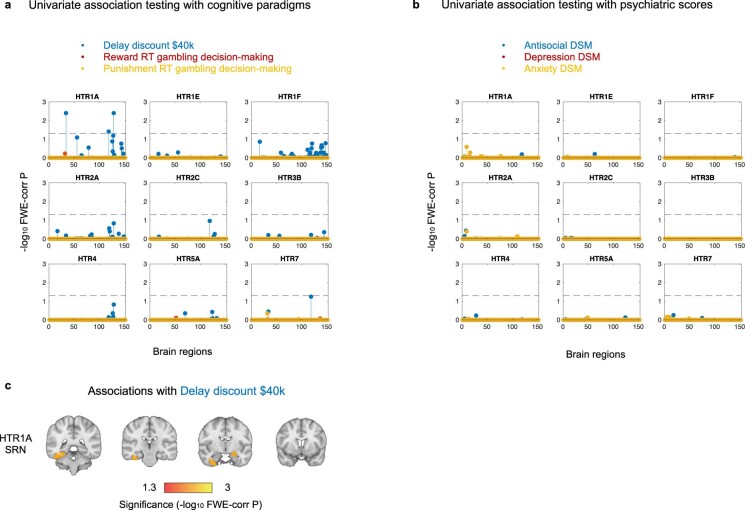
Univariate association testing between single SRNs and mental processes implicated in serotonin function. Related to Fig. 4. **a**) Significance level of permutation analysis of linear models testing the associations between single SRNs and cognitive processes implicated in serotonin function: delay discount (blue), reward decision-making (red), punishment decision making (yellow). **b**) Significance level of permutation analysis of linear models testing the associations between single SRNs and reported psychiatric problems linked to alterations of serotonin functioning: antisocial (blue), depression (red), anxiety (yellow). In both panel **a**) and **b**), statistical significance was assessed with 1,000 block-aware permutations, with FWE-corr for multiple comparisons. Showing -log10 FWE-corr P, corrected across SRNs, mental processes, and two tails. Note: significance levels are reported independently of the positive or negative sign of the association and no correction for multiple testing was applied between the two separate regression models displayed in panel **a**) and **b**). Dashed lines demarcate statistical thresholds. **c**) Brain regions from panel **a**) significantly associated (FWE-corr P < 0.05) between *HTR1A* SRN functional connectivity and delay discount ($40k). Some of these regions, for example the amygdala, are the same regions also reported in the CCA test. Together, these results show weak associations between mental processes previously implicated in serotonin function and *single* SRNs; as opposed to CCA results showing significant associations with *admixtures* of SRNs.

Importantly, we also tested an alternative hypothesis: that single receptors (as opposed to admixtures of receptor types) explain population variability in mental processes. For this analysis we focused on a restricted set of mental processes: delayed reward discounting^11^, reward^15^ and punishment^16^. This has the effect of limiting the multiple comparison problem arising from performing inference testing on multiple behaviors. Using permutation inference testing for multivariate regression, we tested the association of inter-subject differences in delay discounting, reward and punishment (multivariate predictors), with differences in SRN functional connectivity (univariate response). We found that only the association between delay discounting and SRN *HTR1A* functional connectivity in the amygdala survived correction for multiple comparisons (Extended Data Fig. 5a). This association pattern between delay discount and *HTR1A* SRN functional connectivity vaguely resembles results obtained via CCA for *HTR1A* SRN functional connectivity (Extended Data Fig. 4a). Intriguingly, univariate association testing failed to detect associations between delay discount and other *HTR2C* SRNs, as highlighted by the CCA, as would be expected by previous pharmacological manipulations in rodents^43^. We also tested the association of inter-subject differences in antisocial, depression and anxiety problems with differences in SRN functional connectivity. We found no significant association between variation in single SRN functional connectivity and variation in reported psychiatric symptoms (Extended Data Fig. 5b). Although no inference can be drawn by the absence of an association, these results complement results from CCA: the latter showing evidence that admixtures of serotonin receptors may bias inter-subject differences in mental processes previously implicated in serotonin function. Furthermore, CCA results show that different admixtures of serotonin receptors can bias inter-subject differences in distinct mental functions. Hence, these findings provide evidence that spatial distribution and admixture of different SRNs can confer specificity at the brain-wide level not only during serotonin DRN firing or selective manipulation of serotonin availability, but also in the regulation of mental processes.

## Discussion

In this work, we study the large-scale organization of serotonin neuromodulation and the role of different serotonin receptor types in mediating serotonin’s effects on brain-wide activity and behavior. We do this across mice and humans, using a transcriptomic–neuroimaging approach which allows us to characterize brain-wide fMRI signatures of serotonin receptor genes that are known to have heterogeneous spatial expression patterns. We call these fMRI signatures SRNs. In mice, we show that activation of DRN serotonin neurons elicits unique, heterogeneous responses, in both response amplitude and functional connectivity, across different SRNs (Fig. 2). This demonstrates that the spatial distribution of different serotonin receptor types confers functional specificity at the brain-wide level. Crucially, we then show how fluoxetine has bidirectional effects on the DRN serotonin modulation of specific SRNs, as would be predicted from previous pharmacological studies (Fig. 3). In humans, we then ask whether individual differences in the large-scale functional organization of these SRNs underpin population variation in mental functions previously implicated in serotonin regulation. Using population brain imaging from the HCP dataset, we show that individual differences in SRN functional connectivity are organized in two independent modes of population variation (Fig. 4): whilst the first is related to waiting impulsivity, antisocial problems and feelings of aggression, the second is related to a negative bias in reward processing, depression and feelings of panic. These results replicate the established division of the effects of serotonin modulation on human behavior postulated by influential theories of serotonin function^7,8,10^. This suggests that different human behaviors may undergo different serotonin regulation depending on the pattern of serotonin receptor types expressed in the relevant circuits.

Our findings suggest that serotonin brain-wide neuromodulation is a far more complex and multifaceted process than previously thought^29,44^. Driving DRN serotonin neuromodulation through means of ofMRI, and altering synaptic serotonin availability during ofMRI, allowed us to study precisely how different SRNs differ in their responses (network amplitude and functional connectivity changes), and also allowed us to test predictions arising from previous studies on neuropharmacology of serotonin. Different serotonin receptors are expressed with partially overlapping distribution densities in the brain, have different cellular effects and benefit from different affinities to serotonin concentrations^24^. These dissimilarities may explain why we observe a heterogeneity in SRN responses and, in turn, may suggest why different brain areas—characterized by different admixtures of receptor types—may have different sensitivities to serotonin neuromodulation. Indeed, one further possibility is that heterogeneous, brain-wide distribution densities of different serotonin receptor types represent a large-scale principle of organization for serotonin regulation of brain networks and behaviors. Crucially, we tested this hypothesis here via population-level brain imaging in humans. We found that distinct functions ascribed to serotonin, behavioral inhibition and aversive processing, respectively, can be captured into two distinct modes of human population variation, each linked with different fingerprints of SRN functional connectivity across individuals. Indeed, previous work has hinted at the existence of a heterogeneous link between serotonin receptors and behavior. Genetic knockout models of different serotonin receptor genes express contrasting behavioral phenotypes when examined on a number of behavioral paradigms^45^. Serotonin synapses are present brain wide, and are characterized by a complex tapestry of 14 different receptor types which are encoded by seven gene families^3^. As serotonin neuromodulation is systemic, and dramatic differences in patients’ responses to SSRIs exist^3,7^, our findings suggest that inter-individual variation in serotonin regulation may be mediated by differences in the brain-wide functional organization at the serotonin receptor level.

Our findings highlight the link between SRN dynamics and human individual variation in delay discounting, the tendency to choose smaller but immediate rewards over larger but delayed ones. Although the precise significance of serotonin at the computational modeling level is not yet well understood^46^, serotonin is well known to modulate waiting and (im)patience in intertemporal choice in rodents^17–19^. Serotonin has also been implicated in biasing intertemporal decision making during delayed reward discounting in humans, with lower serotonin levels linked to greater temporal discounting^11^. Here, we extend this previous literature by showing that in humans, at the population level, greater temporal discounting was related with greater *HTR1A*, *HTR1F* and *HTR2C* SRN functional connectivity in the amygdala and temporal cortex. The finding that serotonin effect on intertemporal choice is influenced by the regulation of the serotonin 5-HT1A and 5-HT2C receptors is consistent with previous serotonin pharmacological manipulations. Selective 5-HT1A and 5-HT2C receptor antagonist decreases impulsive choice during delay discounting^43,47^. Aligned with this literature, here we found that, in humans, individuals exhibiting lower patience in intertemporal choice preference were those who showed greater *HTR1A*, *HTR2C* and *HTR1F* SRN functional connectivity, specifically in the amygdala and temporal cortex. In rodents, optogenetic inactivation of the amygdala (a region exhibiting high density of 5-HT2C receptors, see Fig. 2b) disrupts intertemporal choice processing, hence playing a causal role during delayed reward task^48^. The results presented here therefore agree with previous work in rodents and suggest that human inter-individual differences in 5-HT1A and 5-HT2C receptor expression in the amygdala may bias humans’ intertemporal choice processing. These results also raise the possibility that 5-HT1F receptors—a still relatively unexplored serotonin receptor type—may play a role in waiting impulsivity. Intriguingly, the plausible link between *HTR2C* SRN and delay discount in a relevant brain region could only be detected by testing the hypothesis that the admixture of serotonin receptors (tested via CCA), and not single receptor types (tested via univariate testing), can explain serotonin modulation of human biases. By studying differences in brain activity and in mental processes previously implicated in serotonin at the human population level, these findings demonstrate that the spatial distribution and admixture of different serotonin receptor types confer functional specificity not only during downstream neuromodulation, as shown here in the mouse experiments, but also in modulating human cognitive biases (impulsivity/patience) in intertemporal decision making.

Our findings also highlight the link between SRNs and population variation in altered reward processing during gambling decision making, independent of the bias in delay discount. This finding is of particular interest because serotonin is known to play a key role in modulating reward both in mice^20^ and in humans^15^. The results show that decreased responsiveness to reward (slower reaction times) was related to greater *HTR1A* and *HTR4* SRN functional connectivity in the parietal cortex and in the insula, putative reward-related brain regions^39,49^. Slower responses to reward (compared with punishment) would lead to enhanced negative or aversive bias in neural responsiveness^50^—an effect that could be interpreted also as a magnified impact of punishment. This result is important because fluoxetine treatments, acting on 5-HT1A and 5-HT4 receptors (as also shown in our mouse results section), are known to improve depression and reward processing^51^. Indeed, it seems that altered serotonin function creates a bias in processing away from positive and towards negative stimuli, with chronic SSRI treatment restoring this processing balance^7,52^. Our findings in humans provide further support to the notion that the spatial distribution and the admixture of serotonin receptors plays a key role in serotonin’s regulation of intertemporal choices and reward processing. This suggests that polymorphisms in serotonin receptors may influence human biases in decision making by altering serotonin interactions with different receptor types.

Our analysis also sheds light on the role of SRN modulation in affective disorders. Whilst individuals showing high impulsivity/low patience during intertemporal choices also reported greater scores in the scale of antisocial personality problems and more intense feelings of aggression (first mode), individuals showing a negative bias to reward during gambling decision making also reported greater levels of depressive symptoms and more intense feelings of panic (second mode). Crucially, both phenotypes, impulsivity–aggression–antisocial^53,54^ and biased reward–panic–depression^52,55^, are compatible with alterations of serotonin function in humans. These findings show that impulsive intertemporal choice processing and biased reward processing are linked with distinct dimensions of psychiatric disorders (antisocial/aggression and depression/panic, respectively) via distinct admixtures of SRNs. By integrating hypothesis- and data-driven approaches across cognition, behavior, affect and psychiatric symptoms in humans, at the population level, we were able to link SRNs with cognitive biases and affective disorders, demonstrating cognitive and psychiatric relevance of two modes of SRN modulation.

Together, the findings reported here show that SRNs have diverse and wide-reaching associations with human mental processes and psychiatric symptoms previously implicated in serotonin function and dysfunction, and that serotonin achieves specific modulation of these processes via (at least two) independent modes of neuromodulation. In other words, at the human population level, serotonin biases different domains of cognition, affect, personality, psychiatric dimensions and social behaviors, via the modulation of distinct brain areas characterized by different admixtures of serotonin receptors. Crucially, we show how this is possible via the neuromodulation mechanisms uncovered in the mouse experiments: both DRN activity and SSRI effects differentially modulate distinct networks of serotonin receptors. These findings demonstrate that the spatial distribution of different serotonin receptor types confers functional specificity at the brain-wide level. They unveil a general principle of how different admixtures of serotonin receptors regulate brain-wide activity and human behavior. Our work provides important insights which complement influential theories on the distinct, perhaps ‘paradoxical’, effects of serotonin on human behavior^7,8,10^. Here, we conceptualize and demonstrate how specificity in serotonin neuromodulation of human behavior can be achieved postsynaptically by leveraging the complex tapestry of serotonin receptors. We show how different admixtures of serotonin receptors, in key brain regions, explain population differences in mental processes previously implicated in serotonin function along two dimensions of SRN modulation: an impulsivity–antisocial dimension, and a biased reward–depression dimension. Crucially, these two dimensions of SRN modulation replicate—and hence explain—established divisions of serotonin effects: serotonin modulates impulsivity and aversive processing^7,8,10^. These findings explain the ‘paradoxical’ effects of serotonin by demonstrating the complementary roles of different brain-wide admixtures of postsynaptic serotonin receptor types on human behavior. Although the diverse effects of serotonin modulation on behavior have been explained hypothetically in terms of actions of distinct serotonin projection systems (DRN versus MRN), recent experimental evidence demonstrates that DRN serotonin neurons play a role in both patience/impulsivity^17–19^ as well as reward/punishment^16,20,21^. Although the role of the MRN in impulsivity and aversive processing can not be ruled out, MRN serotonin neurons are known to regulate anxiety^56^. Hence, this work provides key insights into the roles of different (admixtures of) serotonin receptors in modulating human behavior.

In this work, we exploited the power of cross-species neuroimaging to bridge mechanistic insight in mice with population brain imaging in humans, and unveiled the large-scale functional organization of serotonin neuromodulation. Whilst fMRI is best suited for recording mixed signals of brain-wide activity, transcriptomic maps of serotonin receptor genes (for both the human and the mouse brain) provide the parameters to decode the signals arising from the intricate and diffuse spatial patterns of serotonin receptor types. ofMRI then provides the perfect tool to selectively activate DRN serotonin neurons and, together with SRN maps, allows us to implicate specific patterns of SRN dynamics—otherwise anatomically tangled—in DRN neuromodulation. These fMRI signatures are most likely the result of complex receptor signaling transduction pathways and of cross-neuromodulator cascade events, and hence caution is required in the interpretation of the underlying cellular events^3^. Indeed, seven classes of serotonin receptors exist, most of which are G protein-coupled receptors (GPCRs) (with the exception of 5-HT3 receptors which are ionic channels), and the complexity of their effects scales by the number of proteins with which they interact^23^. These receptors are the target of many therapeutic drugs for treating several psychiatric disorders^3^. Given the widespread prevalence of such conditions worldwide, further efforts are needed to better understand how SRNs play a role in mediating brain-wide neuromodulation, both in health and in disease.

Although the idea of enriching fMRI with molecular information is not new in neuroimaging^57–59^, here we used transcriptomic–neuroimaging mapping to study the effects of an acute pharmacological manipulation during DRN serotonin optogenetic activation. We showed that the SSRI fluoxetine evoked bidirectional changes in the effect of optogenetic manipulation on the SRNs. First, we found that fluoxetine downregulated the optogenetic effect on *Htr1a* and *Htr1b* SRNs. This finding is predicted by previous literature implicating the hyperpolarization of serotonin autoreceptors as a key effect of acute SSRI administration^35^. Second, we found that fluoxetine upregulated DRN activation of *Htr4* SRN. Importantly, the 5-HT4 receptor pathway has previously been shown to play a necessary causal role in the action of fluoxetine^33^. Together, these results show that combining transcriptomics with pharmacological–ofMRI may provide novel neuroimaging markers for serotonin synaptic effects, opening exciting new avenues for personalized medicine.

Our mapping approach between gene expression maps and fMRI has some limitations. First, it is correlational and relies on the fact that different serotonin receptors have partially overlapping distribution densities. Therefore, it is limited by the reliability of characterizing brain-wide gene expression levels and by the co-expression of different receptors^25,26^. The approach used here builds upon established approaches using gene expression maps together with structural MRI to study the biological pathways underlying brain organization^60^. Here, we use the same state-of-the-art brain-wide gene expression maps^25,26^ to study the brain functional organization. Second, our approach is limited by using transcriptomic atlases to infer receptor distributions. PET would arguably be better suited to capture individual differences in receptor density in vivo. Yet, this approach would not allow access to either the spatio-temporal dynamics during neuromodulation, or the sample sizes needed to investigate variation at the population level^57^. Future efforts should therefore be tailored to understand how individual variation in receptor densities may play a role. Third, dorsal raphe neurons are not the only source of brain serotonin. The serotonin MRN is also known to play an important role in serotonin brain regulation by virtue of its complementary projection pattern^56^. This highlights the need to characterize similarities and differences between these two nuclei, their interactions with different serotonin receptor types and their effects on different behaviors.

By reinforcing the importance of cross-species translational research, our results bridge the gap between serotonin manipulations of brain-wide dynamics and population variation in behavior. The findings of this work show that brain-wide admixtures of different serotonin receptor types represent a macroscale principle of organization for serotonin regulation of brain networks and behaviors. Whether other neuromodulatory systems share similar organizational principles remains unknown. Furthermore, whether genetic polymorphisms endow individual variation in SRNs, thus mediating a propensity to developmental and psychiatric disorders or responsiveness to drug treatments, remains unclear. Answering these questions via population medical imaging and genetics may offer novel opportunities for personalized medicine and drug discovery.

## Methods

### Mouse neuroimaging

#### Concurrent ofMRI of DRN serotonin neurons in mice

We used concurrent cerebral blood volume ofMRI data previously published by Grandjean et al.^29^. All experiments and manipulations conformed to the guidelines set by the Animal Care Commission of Switzerland and were covered under the authority of animal permit ZH150/11 given to Isabelle M. Mansuy and ZH263/14 belonging to Bechara J. Saab and in accordance with the UK Animals (Scientific Procedures) Act 1986. We used data from the experiments with ofMRI manipulation of ePet-Cre mice expressing ChR2 in DRN serotonin neurons (*n* = 8, runs = 63), controls expressing eYFP only (*n* = 4, runs = 18) and those ePet-Cre mice treated with fluoxetine before ofMRI (*n* = 6, runs = 18). MRI acquisition, data preprocessing and all surgical procedures are described in detail^29^. Briefly, the light-sensitive ion channel ChR2 was expressed in DRN *Pet-1* serotonin neurons using a cre-dependent adeno-associated virus (AAV-EF1a-DIO-ChR2-EYFP) injected into the DRN of *ePet-cre*^+/−^ mice. Light was delivered through an MRI-compatible optical fiber, implanted in the DRN (medial lateral = 0, anterior posterior = −0.6 mm from Lambda, dorsal ventral = 3.3 mm from the skull), 1–2 weeks post viral infection and at least 1 week before ofMRI. As controls, *ePet-cre*^+/−^ mice underwent the same procedures except that they received a virus lacking ChR2 (AAV-EF1A-DIO-EYFP). fMRI was performed in a 7 T Bruker scanner equipped with a surface coil in mice anesthetized with a mixture of isoflurane (0.5%) and medetomidine (0.1 mg kg^−1^ bolus followed by a constant infusion of 0.2 mg kg^−1^ h^−1^ subcutaneously). Mice were also injected with a paramagnetic iron oxide nanoparticle-based intravascular contrast agent (Endorem, 30 mg kg^−1^ Fe). Each fMRI run consisted of six cycles of 20 s of blue light stimulation at 20 Hz (pulse width = 5 ms, laser power = 40 mW mm^2^) followed by 40 s of rest. Functional images were acquired with a spatial resolution of 0.31 × 0.27 × 0.5 mm^3^ and a temporal resolution of 2 s using a multi-shot gradient echo echo-planar imaging sequence. Data preprocessing was performed using FSL and Analysis of Functional NeuroImages. Anatomical images from each scan session were used to generate a reference template. Linear and non-linear transformations were then estimated between the anatomical images and the reference template. Functional images were temporally realigned, transformed to match the reference template and smoothed using a 0.45-mm^2^ kernel. Time-series were summarized using the Allen Brain Atlas as in ref. ^29^ and sign inverted. The Allen Institute for Brain Science mouse brain atlas was resampled to 90 regions of interest (ROIs) by merging leaves (for example, cortical layers) by branches (for example, cortical area). The nomenclature, and abbreviations for the brain regions, are in accordance with https://atlas.brain-map.org/.

#### Transcriptomic–neuroimaging mapping in mice

We used mouse brain-wide gene expression maps of serotonin receptor genes publicly available from the Allen Brain Institute^25,26^. Gene maps were summarized using the Allen Mouse Brain Atlas as ref. ^29^. Most transcriptomic maps of serotonin receptor genes were available in coronal sections with expression levels across the whole brain (*Htr1a*, *Htr1b*, *Htr2c*, *Htr3a*, *Htr3b*, *Htr5b*). Those that were available in sagittal sections with expression levels for one single hemisphere (*Htr1f*, *Htr2a*, *Htr4*) were thus flipped along the *x* axis and made symmetric. Each serotonin receptor gene map (*Htr1-5*), representing the raw expression level for each gene across the whole brain, was then log_2_-transformed and *Z*-scored across brain regions. Then we used FSL DR (a multiple linear regression method; https://fsl.fmrib.ox.ac.uk/fsl/fslwiki/DualRegression)^27^ to combine serotonin receptor gene maps together with individual ofMRI data. Time-courses from DR-stage 1 were standardized before DR-stage 2 (ref. ^27^). This transcriptomic–neuroimaging approach allows to characterize (1) a time-course representing the network amplitude changes in fMRI activity (DR-stage 1), and (2) a functional connectivity map (DR-stage 2), for each animal, for each serotonin receptor gene. We refer to these signatures as SRNs. A graphical representation of this approach can be found in Fig. 1.

To correct SRN amplitude changes to optogenetic stimulation for baseline differences, we subtracted the average SRN activation during the 40 ofMRI volumes before the first stimulation block from the remaining SRN amplitude time-courses (Extended Data Fig. 2a). We opted for this baseline instead of all the ‘resting’ periods during the stimulation blocks as the elicited SRN amplitude changes are long-lasting way beyond the stimulation phase.

### Statistics and reproducibility

#### Permutation inference testing via general linear models

All inference testing on ofMRI DR-stage 1 and stage 2 outputs (network amplitude changes and functional connectivity maps) was carried out using FSL Permutation Analysis of Linear Models (PALM v.119, https://fsl.fmrib.ox.ac.uk/fsl/fslwiki/PALM^61^). The null distribution was characterized with 1,000 permutations. Statistical significance was established based on family-wise error rate correction (FWE-corr) of *P* values. When testing inferences on SRN temporal responses to optogenetic stimulation (DR-stage 1) (that is, group comparisons: ChR2 versus control animals), one-dimensional (time) threshold-free cluster enhancement was applied^62^. *P* values underwent FWE-corr across time, network testing (SRNs) and two-tails inference (‘greater/smaller amplitude change than’). When testing inferences on brain region functional connectivity changes to optogenetic stimulation (DR-stage 2), no cluster enhancement was applied. *P* values underwent FWE-corr across atlas ROIs, network testing (SRNs) and two-tails inference (‘greater/smaller functional connectivity than’). When testing the effect of fluoxetine, because of the within-subject design, we constrained the permutations to be block-aware, thus allowing permutations only within the same subject across conditions^63^. All statistical significance results are plotted as −log_10_ of FWE-corr *P* and results were deemed significant at FWE-corr *P* < 0.05. All statistical analyses were carried out in MATLAB 2020. The results of these analyses are shown in Figs. 2 and 3 and Extended Data Figs. 2 and 3.

#### Specificity to serotonin receptors in response to DRN ofMRI

We further established the specificity of the transcriptomic–neuroimaging mapping approach to the serotonin receptors alone. To do this, we characterized SRN fluctuations (DR-stage 1) in response to DRN ofMRI after adjusting for 25 non-serotonin receptor maps belonging to other neurochemical modulators: acetylcholine, 16 Chr genes; dopamine, 4 Drd genes; and noradrenaline, 5 Adr genes. We performed DR-stage 1 whilst accounting for 25 non-serotonin receptors. We refer to this approach as residualized DR-stage 1. This approach allows us to establish the ofMRI fluctuations in serotonin receptors after adjusting for the spatial variance of non-serotonin receptors. In other words, residualized DR-stage 1 shows the ofMRI fluctuations that are unique to each serotonin receptor. The same approach was then applied to the other neurotransmitter receptor maps. These analyses were carried out in MATLAB 2020. The results of these analyses are shown in Extended Data Fig. 2.

To further establish specificity, we also replicated the approach previously implemented by Zerbi et al.^31^. Spearman’s partial correlation was used to relate neurotransmitter receptor maps to DRN ofMRI changes (ChR2 group versus controls). To explicitly assess specificity, receptors belonging to a different neuromodulator family were used as covariates of no interest in different partial correlation analyses. As for Zerbi and colleagues, contributions from receptors within the same neuromodulator families were not regressed out because of their strong intrinsic co-expression. Two-tailed significance was assessed with permutation testing and partial correlations were deemed significant at FWE-corr *P* < 0.025. These analyses were carried out in MATLAB 2020. The results of these analyses are shown in Extended Data Fig. 2.

### Human neuroimaging

#### rs-fMRI in the HCP

We used resting-state BOLD fMRI data from *n* = 812 subjects from the HCP, which provides the required ethics and consent needed for study and dissemination, such that no further additional institutional review board approval is required. These are all subjects with complete rs-fMRI data, all healthy adults (aged 22–35 yr, 410 females) scanned on a 3-T Siemens Connectome Skyra. For each subject, four 15-min runs of fMRI time-series data with a temporal resolution of 0.73 s and a spatial resolution of 2-mm isotropic were available. The preprocessing pipeline followed the technique in refs. ^64,65^, and thus will be described only briefly here. Spatial preprocessing was applied using the procedure described in ref. ^66^. We applied structured artefact removal using independent component analysis (ICA) followed by FMRIB’s ICA-based X-noisefier (FIX) from the FSL^67^, which removed more than 99% of the artefactual ICA components in the dataset. We did not use global signal regression. This resulted in 812 subjects, each having 4 rs-fMRI runs of 1,200 time points.

#### Transcriptomic–neuroimaging mapping in humans

We used serotonin receptor gene brain maps from the Allen Human Brain Atlas (AHBA) (*Htr1-7* (ref. ^26^)). The microarray datasets were processed as described in ref. ^68^. Specifically, microarray probes from each of the six donors in the AHBA were initially filtered to retain probes with existing Entrez Gene IDs. The remaining probes were subsequently filtered using the AHBA intensity-based filtering binary indicators, such that the probes for which fewer than 50% of the samples passed the filter were discarded. For every donor, the expression values of multiple probes were then averaged when those probes corresponded to the same gene. These averages were computed in linear space, and the aggregated values were subsequently transformed back to log space using a log_2_ transformation. The resulting gene-by-sample expression matrices were annotated such that the samples were mapped to the structure labels of an atlas parcellation. The atlas labels were assigned to samples on the basis of minimal Euclidean distance in Montreal Neurological Institute coordinate space. To do so, we used an atlas containing 152 cortical and subcortical regions, which was generated by merging the Automated Anatomical Labeling (AAL) cortical atlas^69^ with the five-atlas subcortical^70^, cerebellum^71^, thalamus and striatum^72^, hippocampus subfields^73^ and amygdala^74^ atlases from CoBrALab. The expression of every gene was then averaged over multiple samples with common atlas labels. This was done in linear space before converting back to log space with a log_2_ transformation. Finally, the structure-wise gene expression values were averaged across the two donors in the AHBA that have bilateral sampling (H0351.2001, H0351.2002), resulting in the final gene-by-region expression matrix.

For each of the 812 subjects, separately for each of the four rs-fMRI runs, FSL DR (with variance normalization) was then used to combine (rank-based inverse normalized) brain maps of serotonin receptor genes (*HTR1A*, *HTR1E*, *HTR1F*, *HTR2A*, *HTR2C*, *HTR3B*, *HTR4*, *HTR5A*, *HTR7*) with rs-fMRI data at the voxel-wise level. (Serotonin receptor genes for the human brain are indicated in capital letters to distinguish from those of the mouse brain.) SRN functional connectivity maps were estimated separately for each rs-fMRI dataset and then averaged across the four runs for each subject, resulting in a single functional connectivity map per SRN per subject. Group-average un-threshold SRN maps are made publicly available in NeuroVault.

Although SRN functional connectivity maps are at the voxel-wise level (Fig. 4a), to ease the subsequent statistical analysis, for each SRN we summarized functional connectivity values based on the modified AAL atlas described above. This resulted in a subject × atlas ROIs × SRNs matrix (812 subjects × 152 ROIs × 9 SRNs) which we fed to the following statistical analysis.

#### Behavioral measures in the HCP

We used the restricted behavioral data as provided by the HCP consortium in the CCA. These variables represent a summary of demographic measures present in the HCP sample (full detailed description can be found in ref. ^75^). We then selected a subset of these variables to anchor the results of the brain–behavior covariation analysis and thus to ease results interpretation. These are human mental functions previously implicated in serotonin regulation: delayed reward discounting, affect, personality traits and social behavior (Supplementary Table 1).

### Statistics and reproducibility

#### Permutation inference testing via CCA

To avoid an overdetermined, rank-deficient CCA solution, and to limit the chances of overfitting, a dimensionality reduction step was performed for both brain imaging and behavioral variables. Using the same approach previously applied in ref. ^76^, brain images (SRNs) were reduced using principal component analysis (PCA) into 20 principal components (using the ‘elbow’ rule; variance explained > 60%).

To study whether multiple modes of brain–behavior covariation exist, we used CCA as implemented in ref. ^77^ (https://github.com/andersonwinkler/PermCCA). This allowed us to test whether sets of SRNs were significantly related to behavioral phenotypes. Canonical correlations were estimated in a stepwise manner, removing at each iteration the variance already explained by previous modes of population covariation, while dealing with different numbers of variables in both sides. Importantly, this implementation of CCA also performs residualization of confounds of no interest without introducing dependencies among the observations, which would violate the exchangeability assumption^77^. Here, imaging (20 variables) and behavioral (45 variables) measures were adjusted for 17 confounding variables as previously performed by Smith and colleagues^1^ ((1) acquisition reconstruction software version; (2) average subject head motion during rs-fMRI; (3) weight; (4) height; (5) systolic and (6) diastolic blood pressure; (7) hemoglobin A_1C_; (8) cube-root of total brain volume; (9) cube-root of total intracranial volume, as well as the squared version of measures 2–9 (the first is binary)). Literature shows that CCA results tend to be stable with a large number of subjects in relation to the number of variables^78^. Statistical significance was tested with 10,000 block-aware permutations respecting HCP family-structure^63^ and FWE-corr was applied across all CCA modes. CCA imaging and behavioral cross-loadings were then extracted for all CCA modes deemed significant at FWE-corr *P* < 0.05. The results of these analyses are shown in Fig. 4.

CCA imaging cross-loadings were calculated for all brain regions and all SRNs (Extended Data Fig. 4a)—these were not estimated at the voxel-wise level to preserve the spatial resolution used for statistical analysis. This resulted in two matrices of CCA cross-loadings, one per significant CCA mode, each with a dimension of 152 brain regions by 9 SRNs. To explain the maximum amount of variance, PCAs (with only one single principal component) were performed separately for each of these matrices. Whilst PCA coefficients represent the involvement of each SRN in the mode of covariation (Extended Data Fig. 4b), PCA scores capture the brain correlate (Fig. 4c (thresholded) and Extended Data Fig. 4c (unthresholded)) associated with the CCA imaging cross-loadings across SRNs.

CCA behavioral cross-loadings were calculated for all 45 non-imaging measures (Fig. 4e (showing only cognitive and affective variables) and Extended Data Fig. 4c (showing all variables)). For those variables with high CCA cross-loadings, boxplots of raw scores are shown in Fig. 4f. Separately for each CCA mode, subjects were ranked based on the CCA subject score. Subjects were then divided into a ‘high scoring’ and a ‘low scoring’ group based on the 50th percentile. Boxplots were created using the function *daboxplot* for MATLAB (https://github.com/frank-pk/DataViz).

### Reporting summary

Further information on research design is available in the Nature Portfolio Reporting Summary linked to this article.

## Online content

Any methods, additional references, Nature Portfolio reporting summaries, source data, extended data, supplementary information, acknowledgements, peer review information; details of author contributions and competing interests; and statements of data and code availability are available at 10.1038/s41593-022-01213-3.

## Supplementary information

Supplementary Information Supplementary Table 1.

Reporting Summary

## Data Availability

Mouse ofMRI raw data are publicly available (raw fMRI data: https://openneuro.org/datasets/ds001541/versions/1.1.3; 10.18112/openneuro.ds001541.v1.1.3; preprossed time-series: 10.34973/raa0-5z29; 10.34973/raa0-5z29). Human fMRI and behavioral data are publicly available at https://db.humanconnectome.org/. Transcriptomic data for both the mouse and human brain are publicly available at https://portal.brain-map.org/.

## Code availability

Most of the software and code used in this study are publicly available. Brain image processing and statistical testing were largely carried out with FSL (FMRIB’s Software Library, https://fsl.fmrib.ox.ac.uk/fsl/fslwiki) and MATLAB-based code from FSLNets (https://fsl.fmrib.ox.ac.uk/fsl/fslwiki/FSLNets) and PermCCA (https://github.com/andersonwinkler/PermCCA).
