# Supplementary material for: Serotonin regulation of behavior via large-scale neuromodulation of serotonin receptor networks
Source: Nat Neurosci. 2022 Dec 15;26(1):53–63. doi: 10.1038/s41593-022-01213-3 (PMC9829536; doi:10.1038/s41593-022-01213-3)
Supplement: Supplementary file 1 — Supplementary Table 1. [file 41593_2022_1213_MOESM1_ESM.pdf]

nature neuroscience

Article

https://doi.org/10.1038/s41593-022-01213-3

Serotonin regulation of behavior via large-scale neuromodulation of serotonin receptor networks

In the format provided by the authors and unedited

Supplementary Table 1.

List of selected HCP-variables.

Cognition

Emotion_Task_Face_Acc
Gambling_Task_Reward_Median_RT_Larger
Gambling_Task_Punish_Median_RT_Larger
Flanker_Unadj (inhibition)
CardSort_Unadj (flexibility)
IWRD_TOT (episodic memory, accuracy)
IWRD_RTC (episodic memory, reaction time correct responses)
DDisc_SV_1mo_200 (delay discount, $200)
DDisc_SV_6mo_200
DDisc_SV_1yr_200
DDisc_SV_3yr_200
DDisc_SV_5yr_200
DDisc_SV_10yr_200
DDisc_SV_1mo_40K (delay discount, $40k)
DDisc_SV_6mo_40K
DDisc_SV_1yr_40K
DDisc_SV_3yr_40K
DDisc_SV_5yr_40K
DDisc_SV_10yr_40K

Personality

NEOFAC_A
NEOFAC_O
NEOFAC_C
NEOFAC_N
NEOFAC_E
SSAGA_Times_Used_Hallucinogens
SSAGA_Times_Used_Cocaine

Affect

LifeSatisf_Unadj
MeanPurp_Unadj
PosAffect_Unadj
AngAffect_Unadj
AngHostil_Unadj
AngAggr_Unadj
FearAffect_Unadj
Sadness_Unadj
SelfEff_Unadj
DSM_Antis_Raw
DSM_Depr_Raw
DSM_Anxi_Raw
ASR_Anxd_Raw

Social behaviour

Friendship_Unadj
Loneliness_Unadj
PercHostil_Unadj
PercReject_Unadj
EmotSupp_Unadj
InstruSupp_Unadj
