# Supplementary material for: Serotonin regulation of behavior via large-scale neuromodulation of serotonin receptor networks
Source: Nat Neurosci. 2022 Dec 15;26(1):53–63. doi: 10.1038/s41593-022-01213-3 (PMC9829536; doi:10.1038/s41593-022-01213-3)
Supplement: Supplementary file 2 — Reporting Summary [file 41593_2022_1213_MOESM2_ESM.pdf]

nature portfolio

Corresponding author(s): Piergiorgio Salvan, PhD

Last updated by author(s): Oct 12, 2022

Reporting Summary

Nature Portfolio wishes to improve the reproducibility of the work that we publish. This form provides structure for consistency and transparency in reporting. For further information on Nature Portfolio policies, see our Editorial Policies and the Editorial Policy Checklist.

Statistics

For all statistical analyses, confirm that the following items are present in the figure legend, table legend, main text, or Methods section.

n/a	Confirmed	
☐	☒	The exact sample size (*n*) for each experimental group/condition, given as a discrete number and unit of measurement
☐	☒	A statement on whether measurements were taken from distinct samples or whether the same sample was measured repeatedly
☐	☒	The statistical test(s) used AND whether they are one- or two-sided<br>*Only common tests should be described solely by name; describe more complex techniques in the Methods section.*
☐	☒	A description of all covariates tested
☐	☒	A description of any assumptions or corrections, such as tests of normality and adjustment for multiple comparisons
☐	☒	A full description of the statistical parameters including central tendency (e.g. means) or other basic estimates (e.g. regression coefficient) AND variation (e.g. standard deviation) or associated estimates of uncertainty (e.g. confidence intervals)
☐	☒	For null hypothesis testing, the test statistic (e.g. *F*, *t*, *r*) with confidence intervals, effect sizes, degrees of freedom and *P* value noted<br>*Give P values as exact values whenever suitable.*
☒	☐	For Bayesian analysis, information on the choice of priors and Markov chain Monte Carlo settings
☐	☒	For hierarchical and complex designs, identification of the appropriate level for tests and full reporting of outcomes
☐	☒	Estimates of effect sizes (e.g. Cohen's *d*, Pearson's *r*), indicating how they were calculated

Our web collection on statistics for biologists contains articles on many of the points above.

Software and code

Policy information about availability of computer code

Data collection	Not applicable.
Data analysis	Most of the software and code used in this study are publicly available. Brain image processing and statistical testing was largely carried out with FSL (FMRIB’s Software Library, v6, https://fsl.fmrib.ox.ac.uk/fsl/fslwiki), MATLAB (v2020), MATLAB-based code from FSLNets (v0.6.3, https://fsl.fmrib.ox.ac.uk/fsl/fslwiki/FSLNets) and PermCCA (vApril2021, https://github.com/andersonwinkler/PermCCA).

For manuscripts utilizing custom algorithms or software that are central to the research but not yet described in published literature, software must be made available to editors and reviewers. We strongly encourage code deposition in a community repository (e.g. GitHub). See the Nature Portfolio guidelines for submitting code & software for further information.

Data

Policy information about availability of data

All manuscripts must include a data availability statement. This statement should provide the following information, where applicable:

- Accession codes, unique identifiers, or web links for publicly available datasets
- A description of any restrictions on data availability
- For clinical datasets or third party data, please ensure that the statement adheres to our policy

The data were collected by the Human Connectome Project, the Allen Brain Project, and by the authors of Grandjean et al., (NatComm) 2019.
Mouse ofMRI raw data are publicly available (raw fMRI data: https://openneuro.org/datasets/ds001541/versions/1.1.3; prepossed time-series: http://dx.doi.org/10.34973/raa0-5z29). Human fMRI and behavioural data are publicly available at https://db.humanconnectome.org/. Transcriptomic data for both the mouse and human brain are publicly available at https://portal.brain-map.org/.

Field-specific reporting

Please select the one below that is the best fit for your research. If you are not sure, read the appropriate sections before making your selection.

☐ Life sciences ☒ Behavioural & social sciences ☐ Ecological, evolutionary & environmental sciences

For a reference copy of the document with all sections, see nature.com/documents/nr-reporting-summary-flat.pdf

Behavioural & social sciences study design

All studies must disclose on these points even when the disclosure is negative.

Study description	The study uses quantitative methods (human: quantitative cross-sectional, mouse: quantitative experimental).
Research sample	The data from this study involved existing datasets.<br>Human: 812 individuals from the Human Connectome Project were used for the human study sample (aged 22–35 years, 410 females). The sample is representative of the young adult healthy US population. We chose this sample precisely for this reason and for the complete cognitive characterisation.<br>Mouse: we used data from Grandjean et al., 2019 (NatComm). We used data from the experiments with ofMRI manipulation of ePet-Cre mice expressing channelrhodopsin-2 (ChR2) in DRN serotonin neurons (N = 8, runs = 63), controls expressing eYFP only (N = 4, runs = 18), and those ePet-Cre mice treated with fluoxetine prior to ofMRI (N = 6, runs = 18).
Sampling strategy	We refer the reader to the publication Barch et al., 2014 (NeuroImage) for details on the sampling strategy of the Human Connectome Project.
Data collection	Procedures for data collection in the Human Connectome Project are detailed in the publication Barch et al., 2014 (NeuroImage).
Timing	Timing of data collection in the Human Connectome Project are detailed in the publication Barch et al., 2014 (NeuroImage).
Data exclusions	812 individuals from the Human Connectome Project with complete rsfMRI data and non-imaging variable were used for the human study sample (aged 22–35 years, 410 females).
Non-participation	Please see Barch et al., 2014 (NeuroImage) for details on the human part of this study, and Grandjean et al., 2019 (NatComm) for details on the mouse part of this study.
Randomization	Not relevant.

Reporting for specific materials, systems and methods

We require information from authors about some types of materials, experimental systems and methods used in many studies. Here, indicate whether each material, system or method listed is relevant to your study. If you are not sure if a list item applies to your research, read the appropriate section before selecting a response.

Materials & experimental systems

n/a	Involved in the study	
☒	☐	Antibodies
☒	☐	Eukaryotic cell lines
☒	☐	Palaeontology and archaeology
☐	☒	Animals and other organisms
☐	☒	Human research participants
☒	☐	Clinical data
☒	☐	Dual use research of concern

Methods

n/a	Involved in the study	
☒	☐	ChIP-seq
☒	☐	Flow cytometry
☐	☒	MRI-based neuroimaging

Animals and other organisms

Policy information about studies involving animals; ARRIVE guidelines recommended for reporting animal research

Laboratory animals	B6.Cg-Tg(Fev-cre)1Esd/J (ePet-cre mice; RRID:IMSR_JAX:012712), male and female, 8 to 16 weeks of age.
Wild animals	n/a
Field-collected samples	n/a
Ethics oversight	Please see Grandjean et al., 2019 (NatComm) for the original statement on ethics oversight.

Note that full information on the approval of the study protocol must also be provided in the manuscript.

Human research participants

Policy information about studies involving human research participants

Population characteristics	Please see Barch et al., 2014 (NeuroImage) for details.
Recruitment	Please see Barch et al., 2014 (NeuroImage) for details.
Ethics oversight	Please see Barch et al., 2014 (NeuroImage) for the original statement on ethics oversight.

Note that full information on the approval of the study protocol must also be provided in the manuscript.

Magnetic resonance imaging

Experimental design

Design type	Resting-state fMRI for humans. Block design for mice optogenentic-fMRI.
Design specifications	Humans rs-fMRI: 4 runs of 1200 fMRI volumes.<br>Mouse ofMRI: 6 blocks per run spaced 40 seconds, 1 or 3 runs per session.
Behavioral performance measures	n/a

Acquisition

Imaging type(s)	Functional, structural.
Field strength	Human MRI: 3T. Mouse MRI: 7T.
Sequence & imaging parameters	Human MRI: TR = 720 ms, echo time = 33.1 ms, multiband factor = 8, flip angle = 52 degrees, field of view = 208x180 mm (matrix = 104 x 90), 2x2x2 isotropic voxels with 72 slices, alternated LR/RL phase encoding.<br>Mouse MRI: multi-shot gradient echo EPI, field of view 20 x 17.5 mm, slice thickness 0.5 mm, slice gap 0.15 mm, 14 slices, 2 segments, TR 1000 ms. TE 5.6 ms, FA 90°, matrix 64 x 64, bandwidth 250000 Hz, 360 or 720 repetitions.
Area of acquisition	Whole brain scan.

Diffusion MRI ☒ Used ☐ Not used

Parameters: Human DW-MRI: The spatial resolution was 1.25 mm isotropic, TR was 5500 ms, TE was 89.50 ms, the b-values were 1000, 2000, and 3000 s/mm2, and
the total number of diffusion sampling directions was 90, 90, and 90 for each of the shells, in addition to 6 b0 images.

Preprocessing

Preprocessing software	We refer the reader to the original articles (and related reporting summaries) for a detailed description: human MRI preprocessing - Smith et al., 2015 (NatNeurosci); and human MRI preprocessing - Grandjean et al., 2019 (NatComm). A summary can be found in the article methods section.
Normalization	We refer the reader to the original articles (and related reporting summaries) for a detailed description: human MRI preprocessing - Smith et al., 2015 (NatNeurosci); and human MRI preprocessing - Grandjean et al., 2019 (NatComm). A summary can be found in the article methods section.
Normalization template	We refer the reader to the original articles (and related reporting summaries) for a detailed description: human MRI preprocessing - Smith et al., 2015 (NatNeurosci); and human MRI preprocessing - Grandjean et al., 2019 (NatComm). A summary can be found in the article methods section.
Noise and artifact removal	We refer the reader to the original articles (and related reporting summaries) for a detailed description: human MRI preprocessing - Smith et al., 2015 (NatNeurosci); and human MRI preprocessing - Grandjean et al., 2019 (NatComm). A summary can be found in the article methods section.
Volume censoring	Scrubbing was not performed on any of these datasets.

Statistical modeling & inference

Model type and settings	Human: A brain-behaviour covariation was tested using canonical correlation analysis with permutation inference testing whilst respecting HCP family-structure using block-aware permutations.<br>Mouse: A difference in the response to photostimulation between wild-type mice vs. transgenic mice was tested via a general linear model with permutation inference testing. The effect of fluoxetine on mice response to photostimulation was tested via a general linear model with permutation inference testing whilst respecting subject-structure using block-aware

permutations.

Effect(s) tested

Human: A brain-behaviour covariation was tested using canonical correlation analysis (CCA) with permutation inference testing whilst respecting HCP family-structure using block-aware permutations.
Mouse: A difference between wild-type mice vs. transgenic mice in the response to photostimulation was tested via a general linear model with permutation inference testing. The effect of fluoxetine on mice response to photostimulation was tested via a general linear model with permutation inference testing whilst respecting subject-structure using block-aware permutations.

Specify type of analysis: ☐ Whole brain ☐ ROI-based ☒ Both

Anatomical location(s)

Human:
Brain atals: we used a novel atlas containing 152 cortical and subcortical regions, which was generated by merging the AAL cortical atlas with the 5-atlas subcortical, cerebellum, colin27 thalamus and striatum, hippocampus subfields, and amygdala atlases from CoBrALab.
Serotonin receptor networks (SRNs): HTR1A, HTR1E, HTR1F, HTR2A, HTR2C, HTR3B, HTR4, HTR5A, HTR7.
Mouse:
Brain atlas: the Allen Institute for Brain Science (AIBS) mouse brain atlas was resampled to 90 regions-of-interest by merging leafs (e.g., cortical layers) by branches (e.g., cortical area).
Serotonin receptor networks (SRNs): Htr1a, Htr1f, Htr1b, Htr2a, Htr2c, Htr3a, Htr3b, Htr4, Htr5b.

Statistic type for inference
(See Eklund et al. 2016)

Human: Non-parametric permutation testing with block-aware permutations.
Mouse: Non-parametric permutation testing with block-aware permutations and cluster correction.

Correction

Human: Statistical significance was tested with 10,000 block-aware permutations respecting HCP family-structure and a family wise error (FWE)-correction was applied across all CCA modes.
Mouse: Statistical significance was tested with 1,000 block-aware permutations and FWE-correction. A one-dimensional (time) threshold-free cluster enhancement (TFCE) was applied when testing group differences on SRN temporal responses to optogenetic stimulation. P-values were FWE-corrected across time, network tested (SRNs), and two-tails inference.

Models & analysis

n/a	Involved in the study
☐	☒ Functional and/or effective connectivity
☒	☐ Graph analysis
☐	☒ Multivariate modeling or predictive analysis

Functional and/or effective connectivity

Functional connectivity metrics for SRNs were computed using FSL dual regression (https://fsl.fmrib.ox.ac.uk/fsl/fslwiki/DualRegression).

Multivariate modeling and predictive analysis

Human: In order to avoid an overdetermined, rank-deficient CCA solution, and to limit the chances of overfitting, a dimensionality reduction step was performed prior to CCA using principal component analysis (PCA) whilst retaining >60% of variance (PCAs number identified via 'elbow' rule). In order to study whether significant modes of brain-behaviour covariation exist whilst adjusting for confounds of no interest, we used CCA as implemented in Winkler et al. (https://github.com/andersonwinkler/PermCCA).
